# Detecting Cognitive Impairment in Idiopathic Intracranial Hypertension Using Ocular Motor and Neuropsychological Testing

**DOI:** 10.3389/fneur.2021.772513

**Published:** 2021-11-16

**Authors:** Wendy Wang, Meaghan Clough, Owen White, Neil Shuey, Anneke Van Der Walt, Joanne Fielding

**Affiliations:** ^1^Department of Neuroscience, Central Clinical School, Monash University, Melbourne, VIC, Australia; ^2^Department of Neurology, Alfred Hospital, Melbourne, VIC, Australia; ^3^Royal Victorian Eye and Ear Hospital, Melbourne, VIC, Australia

**Keywords:** idiopathic intracranial hypertension, cognitive impairments, ocular motor, neuropsychology, inhibitory control, visual processing

## Abstract

**Objective:** To determine whether cognitive impairments in patients with Idiopathic Intracranial Hypertension (IIH) are correlated with changes in visual processing, weight, waist circumference, mood or headache, and whether they change over time.

**Methods:** Twenty-two newly diagnosed IIH patients participated, with a subset assessed longitudinally at 3 and 6 months. Both conventional and novel ocular motor tests of cognition were included: Symbol Digit Modalities Test (SDMT), Stroop Colour and Word Test (SCWT), Digit Span, California Verbal Learning Test (CVLT), prosaccade (PS) task, antisaccade (AS) task, interleaved antisaccade-prosaccade (AS-PS) task. Patients also completed headache, mood, and visual functioning questionnaires.

**Results:** IIH patients performed more poorly than controls on the SDMT (*p*<* 0.001*), SCWT (*p* = *0.021*), Digit Span test (*p*< *0.001*) and CVLT (*p* = *0.004*) at baseline, and generated a higher proportion of AS errors in both the AS (*p*< *0.001*) and AS-PS tasks (*p* = *0.007*). Further, IIH patients exhibited prolonged latencies on the cognitively complex AS-PS task (*p* = *0.034*). While weight, waist circumference, headache and mood did not predict performance on any experimental measure, increased retinal nerve fibre layer (RNFL) was associated with AS error rate on both the block *[F*_(3, 19)_=*3.22, B* = *0.30, p* = *0.022]* and AS-PS task *[F*_(3, 20)_ = *2.65, B* = *0.363, p* = *0.013]*. Unlike ocular motor changes, impairments revealed on conventional tests of cognition persisted up to 6 months.

**Conclusion:** We found multi-domain cognitive impairments in IIH patients that were unrelated to clinical characteristics. Marked ocular motor inhibitory control deficits were predicted by RNFL thickness but remained distinct from other cognitive changes, underscoring the significance of visual processing changes in IIH.

## Introduction

Idiopathic intracranial hypertension (IIH) is characterised by increased cerebrospinal fluid pressure with an unclear aetiology. IIH affects predominantly young women, and is associated with serious consequences, including loss of vision, disabling headache, and loss of employment (1). Evidence is emerging that IIH patients also experience a range of cognitive impairments (2–7) that, despite effects on decreased quality of life and poor treatment outcomes (5), remain under-recognised and poorly understood. Whether cognitive changes are independent or a consequence of other features of the disorder, such as changes in visual processing, headache, mood disorders, weight and medication, is unknown.

Here we assessed cognitive changes in IIH using conventional neuropsychological measures and novel ocular motor tasks that examine visual processing changes associated with saccade generation (8). Ocular motor tasks were the simple prosaccade (PS) task, which requires a gaze shift toward a suddenly-appearing stimulus, reflecting simple sensorimotor processing, and the more complex antisaccade (AS) task that additionally implicates cognitive networks required to inhibit a saccade toward a suddenly-appearing stimulus and then move the eyes in the opposite direction (9). Task demands were modified using the interleaved AS-PS task that recruits a broader cognitive network, enabling assessment of the interaction between changes in visual processing and cognitive function more broadly (10). Relationships were assessed between cognitive impairments and common co-morbid IIH features, such as headache, mood, weight, waist circumference and visual processing changes.

## Methods

### Participants

Twenty-two patients with probable (*n* = *2*) or definite (*n* = *20*) IIH based on revised diagnostic criteria proposed by Friedman et al. (11) were recruited from a tertiary Neuro-Ophthalmology clinic in Melbourne, Australia. Fourteen IIH patients completed testing at 3 months and five at 6 months, which was limited by Covid-19.

To decrease additional confounding factors, or barriers to testing, patients were excluded if they had severe vision-threatening IIH, were pregnant, or had co-existing severe neurological or mental health disorders (such as neurological deficits resulting in difficulty writing or decreased concentration due to hallucinations).

All IIH patients underwent comprehensive neurological and neuro-ophthalmic assessment, including tests of visual acuity, perimetry and optical coherence tomography (OCT). Baseline patient characteristics are displayed in Table 1.

**Table 1:** Demographic information for all participants.

	**IIH**	**OM controls**	**NP controls**
	**mean (SD)**	**mean (SD)**	**mean (SD)**
	***n = 22***	***n = 12***	***n = 22***
Female/male	22/0	12/0	22/0
Age/distribution	27.32/19–46	23.58/21–30	31.43/20–42
Mean NART FSIQ/distribution idiopathic intracranial hypertension	118/97–128		116/109–128
Duration (months)	2.7 (1.4)		
CSF opening pressure (cm)	29.5 (4.8)		
Weight (kg)	103.6 (33.3)		
Waist (cm)	107.9 (20.9)		
Headache/Visual symptoms (%)	91/18		
Acetazolamide/Topiramate (%)	59/23		
VA right/left (LogMAR)	−0.1 (0.1)/ −0.1 (0.1)		
HVF 30–2 right/left (PSD)	2.7 (2.5)/3.1 (2.9)		
RNFL right/left (μm)	125.1 (46.9)/119.9 (36.5)		

*OM, Ocular Motor; NP, Neuropsychology; NART FSIQ, National Adult Reading Test Full Scale Estimated IQ; VA, Visual Acuity; OD, Right; OS, Left; HVF, Humphrey Visual Fields; PSD, Pattern Standard Deviation; RNFL, Retinal Nerve Fibre Layer*.

Healthy control data were sourced from existing ocular motor and neuropsychology databases (12). Twelve ocular motor and twenty-two neuropsychology control datasets were included. IIH and ocular motor control groups were matched for age and sex. IIH and neuropsychology control groups were matched for age, sex and intelligence as estimated by the National Adult Reading Test (NART)(13).

### Standard Protocol Approvals, Registrations, and Patient Consents

Ethics approval was granted by the Alfred Health Research Ethics Committee. Participants provided written informed consent prior to participation in the study in accordance with the declaration of Helsinki.

### Data Availability Statement

Relevant data not published within the article can be made available by the corresponding author on reasonable request.

### Equipment, Stimuli, and Procedures

All testing took place at the Central Clinical School in the Alfred Centre at Monash University.

### Clinical Assessments and Optical Coherence Tomography

Visual assessments were completed by qualified orthoptists and neuro-ophthalmologists.

Perimetry was conducted using a Humphrey Visual Field analyser, set at 30-2. OCT was performed in all participants using Zeiss Cirrus technology according to published standards (14). Scans were acquired on the same day as clinical and cognitive assessments at a single centre, on a single machine using semi-automatic settings, by a single operator. Testing was performed undilated in a brightly lit room. Quality control using the OSCAR-IB criteria (15) was applied and all scans were included for analysis. RNFL thickness was derived from Zeiss Windows 7 Version 11 software.

### Questionnaires

Patient rated outcomes included assessments of headache (Headache Impact Test 6: HIT-6) (16), anxiety (Penn State Worry Questionnaire: PSWQ) (17), depression (Patient Health Questionnaire 9: PHQ-9) (18) and visual functioning (National Eye Institute Visual Functioning Questionnaire 25: NEI-VFQ 25) (19).

### Conventional Cognitive Assessments

The Symbol Digit Modalities Test (SDMT) assesses information processing speed (20), Stroop Colour and Word Test (SCWT) provides a marker of inhibition of cognitive interference (21), and both the Digit Span (22) and California Verbal Learning Test (CVLT) (23) assess working memory.

### Ocular Motor Assessments

Ocular motor tasks were conducted in a darkened room, with participants seated on a chair adjusted for height, 950 mm from a 60 Hz LCD monitor (resolution: 1,920 ×1,080). Task stimuli were green, blue and purple crosses presented on a black background. Horizontal eye movements were recorded using an Eyelink 1,000 plus dark pupil video-oculography system, which has high resolution (noise limited at < 0.01 degrees) and an acquisition rate of 1,000 Hz. Saccadic latencies (ms) and error (%) were recorded for all tasks, which were compared to control data.

### Block Prosaccade (PS) Task

**Figure 1:**
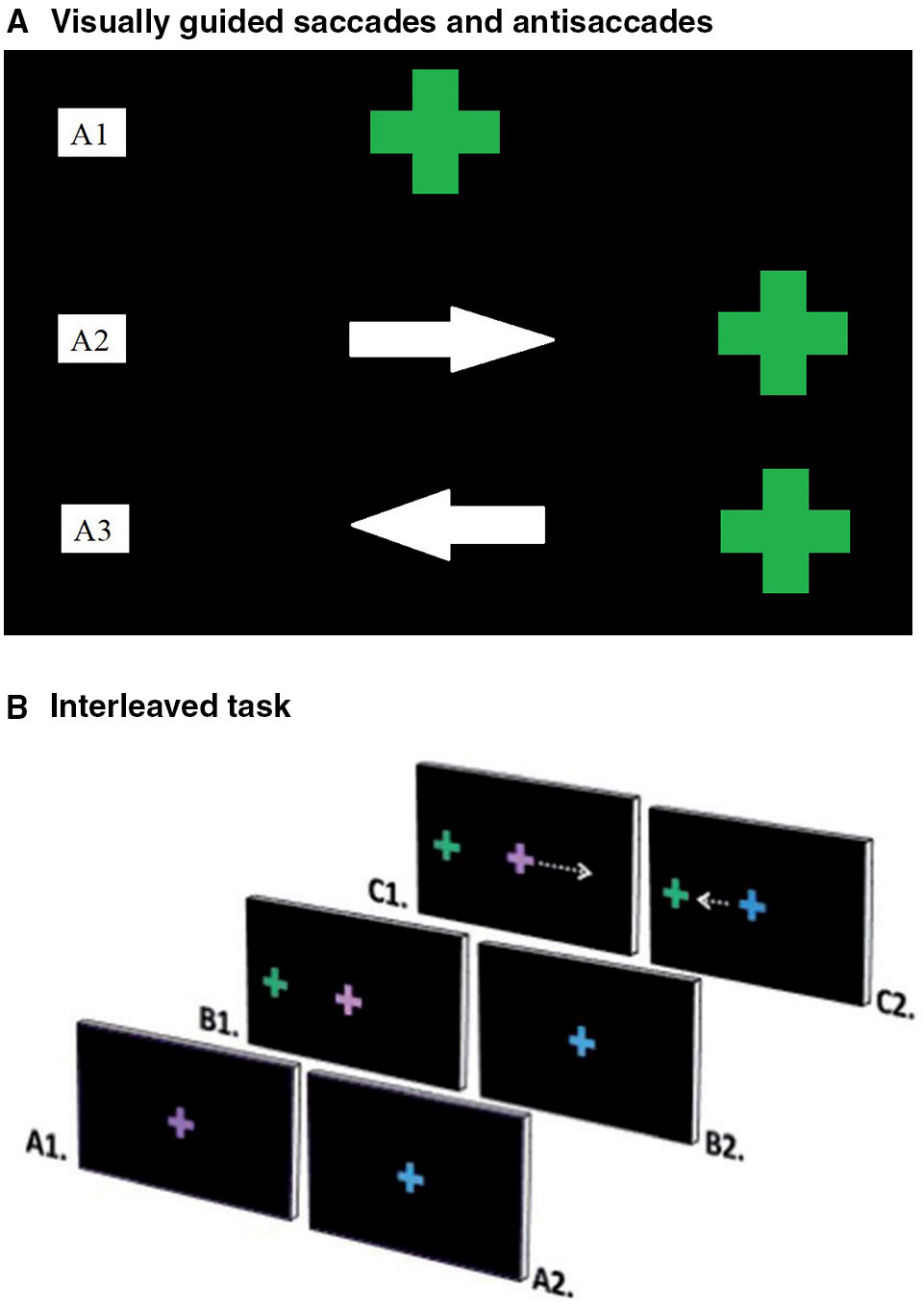
**(A)** Ocular motor tasks. **(A)** Visually guided saccades and Antisaccades. Visually guided saccades assessed baseline visual pathway integrity (24). Participants fixated on a central green cross **(A1)**, which moved right or left and back to centre. They were instructed to follow the cross with their eyes as accurately as possible **(A2)**. Antisaccades assessed inhibitory control; the ability to inhibit an automatic response, in favour of performing a goal-oriented response requiring more effort (8). Participants fixated on a central green cross **(A1)** that disappeared and reappeared either to the left or right on screen. Participants were instructed *not* to look at the green cross and instead to look at the mirror opposite location to the green cross when it reappeared **(A3)**. **(B)** Interleaved task. The interleaved task assessed attention and working memory by presenting a stimulus, which cued participants to respond in one of two ways (25). Participants fixated on the centre of the screen, where either a purple **(A1)** or blue **(A2)** cross appeared. A green cross then appeared peripherally **(B1)**. Participants performed a prosaccade or antisaccade movement, depending on the colour of the initial cross. An antisaccade was cued by the purple cross **(C1)** and a prosaccade by the blue cross **(C2)**. Repeat consecutive trials were considered easier than switching tasks between trials.

The PS task consisted of 96 randomly presented trials completed in a single block. Participants initially fixated a centrally located green cross and were instructed to follow the cross with their eyes as it moved to one of four peripheral locations (5 or 10 degrees to the right or left of centre, presented for 1,500 ms) and back to centre (Figure 1A). To reduce anticipatory responses, the central cross was presented for 1,000, 1,250, or 1,500 ms.

### Block Antisaccade (AS) Task

The AS task consisted of 24 randomly presented trials in 2 blocks. Participants initially fixated a centrally located green cross. After either 1,000, 1,250 or 1,500 ms, the central cross disappeared and re-appeared at either 5 or 10 degrees left or right of centre for 1,500 ms. Participants were instructed to look at the mirror opposite position of the peripherally located cross, rather than directly at the cross (Figure 1A).

### Interleaved Antisaccade-Prosaccade (AS-PS) Task

The AS-PS task consisted of 16 PS trials and 16 AS trials, presented in 3 blocks in a pseudo-random order. Prior to testing, participants performed a practise block of 5 PS trials and 5 AS trials. During the task, participants initially fixated a centrally located blue or purple cross for 1,000, 1,250, or 1,500 ms. The central cross then disappeared and a green cross appeared either 5 or 10 degrees to the left or right of centre for 1,500 ms (Figure 1B). A blue central cross indicated that participants should look toward the peripheral green cross (PS). A purple central cross indicated that participants should look at the mirror opposite position of the green cross (AS). The second of two consecutive trials of the same trial type (e.g., PS followed by PS) was classified as a repeat trial, while the second of two consecutive trials of different types (e.g., PS followed by AS) was classified as a switch trial. The first trial of each block was excluded from repeat or switch trial type analyses, since there was no preceding trial.

### Data Analysis

A custom Matlab program was utilised to analyse eye movement data. Saccade latency (ms) was calculated from a monocular eye trace as the time difference between the onset of a target and onset of a saccade. The onset of a saccade was determined by a visual change in the baseline saccade trace and calculated using a velocity criterion of 30° per second. An error, calculated as a percentage of the total number of trials, was recorded if participants generated a saccade of >1.5 degrees in the wrong direction (e.g., PS movement during an AS trial). Errors were not applicable to PS block trials. Trials that involved an error, blink artefact, absent response, or fixation loss of 2 degrees from the central target, were excluded from latency analysis.

All data were analysed using IBM SPSS Statistics Version 26. Due to violations of normality using Shapiro-Wilks analyses, group comparisons were conducted using Mann-Whitney *U*. Mann-Whitney *U* tests separately compared IIH and control groups for all cognitive assessments. For the AS-PS task, switch cost (differences in performance between non-switch and switch trials) could not be determined due to the high proportion of excluded trials for the IIH cohort as a result of error. Multivariate linear regressions were performed to determine whether certain clinical variables (weight, waist circumference, depression, anxiety, headache, RNFL) predicted cognitive performance (either neuropsychological test or ocular motor). Correction for multiple comparisons were performed using the Benjamini-Hochberg methodology (26), with a false discovery rate of 80%. All significant findings and p-values presented survived corrections. Those that did not have been stated as non-significant.

## Results

All 22 participants were phenotypically typical of IIH and were tested at a mean of 2.7 months from diagnosis [*standard deviation (SD)* = *1.4*]. Thirteen patients were managed with acetazolamide, 5 with topiramate and 4 were not on medications for IIH. Additional clinical characteristics and baseline demographics are shown in Table 1. Table 2 summarises group means and standard deviations for all tasks.

**Table 2:** Ocular motor and neuropsychology means and standard deviations.

	**Control**	**IIH baseline**	**IIH 3-month**	**IIH 6-month**
	**mean (SD)**	**mean (SD)**	**mean (SD)**	**mean (SD)**
	***n = 12***	***n* = 22**	***n* = 14**	***n* = 5**
**Ocular motor**				
Prosaccade latencies (ms)				
PS block	213.22 (26.24)	205.22 (21.27)	206.14 (19.08)	202.34 (14.36)
Repeat PS	230.79 (36.07)	211.37 (28.36)	217.17 (41.13)	213.18 (19.23)
Switch PS	237.03 (51.99)	242.12 (59.64)	236.86 (48.51)	216.96 (20.28)
Prosaccade error rate (%)				
Repeat PS	1.00 (2.11)	5.22 (19.74)	0.36 (1.34)	0.00 (0.00)
Switch PS	3.20 (3.16)	6.78 (16.47)	2.00 (2.08)	0.00 (0.00)
Antisaccade latencies (ms)				
AS block	312.88 (33.96)	335.56 (41.78)	354.49 (62.78)	317.31 (32.52)
Repeat AS	305.85 (43.00)	346.26 (60.62)	343.06 (50.70)	375.80 (98.72)
Switch AS	300.38 (48.53)	349.32 (63.50)^*^	338.38 (49.33)	329.49 (38.76)
Antisaccade error rate (%)				
AS block	6.06 (8.25)	34.33 (21.47)^*^	22.05 (22.22)^*^	25.83 (38.00)
Repeat AS	5.22 (9.12)	29.49 (23.96)^*^	17.70 (26.28)	32.17 (27.85)
Switch AS	9.60 (8.88)	22.26 (22.81)	15.14 (17.62)	18.40 (16.32)
**Neuropsychology**				
SDMT (score)	74.59 (12.27)	47.90 (14.02)^*^	51.08 (13.84)^*^	57.40 (12.90)^*^
SCWT (*t*-score)	53.77 (12.20)	45.73 (6.13)^*^	42.36 (5.27)^*^	39.40 (7.27)^*^
Digit Span (score)	12.68 (3.05)	9.55 (2.22)^*^	8.79 (2.29)^*^	8.20 (2.28)^*^
CVLT (*t*-score)	43.05 (10.71)	36.09 (17.49)^*^	N/A	N/A

* *Sig p < 0.05, compared to controls*.

*PS, prosaccade; AS, antisaccade; SDMT, symbol digit modalities test; SCWT, Stroop colour and word test; CVLT, California Verbal Learning Test*.

### Questionnaires

At baseline, responses from the HIT-6 indicated that participants generally experienced prominent headaches, with a mean score of 57.8 (*SD*=*11.6, range* = *36–76, prominent if score* >*50*) (16). The PSWQ and PHQ-9 revealed moderate levels of anxiety and depression in the IIH cohort, with mean scores of 51.1 (*SD*=*14.3, range* = *32–75, moderate if score 40–59*) and 11.8 (*SD*=*15.0, range* = *0–75, moderate if score 10–14*), respectively (17, 18).

Headache, anxiety and depression in the IIH group remained statistically comparable to baseline at 3 and 6-months; HIT-6 (*mean* = *55, SD* = *10.9, range* = *38–78*), PSWQ (*mean* = *55, SD* = *14.7, range* = *28–80*), and PHQ-9 (*mean* = *9.3, SD* = *6.1, range* = *2–20*).

IIH patients had no significant impairments in perceived visual functioning based on responses from the NEI-VFQ 25 questionnaire (19) at any time-point.

### Neuropsychological Assessments

At baseline, IIH patients performed more poorly than healthy controls on the SDMT (*U* = *27, p*< *0.001*), SCWT (*U* = *143.5, p* = *0.021*) Digit Span (*U* = *94, p*< *0.001*), and CVLT (*U* = *114, p* = *0.004*). For IIH patients, performance remained significantly poorer than controls at 3-months (*SDMT: U* = *28, p*< *0.001, Stroop: U* = *65.5, p* = *0.003, Digit Span: U* = *43.5, p*< *0.001*) and 6-months (*SDMT: U* = *19.5, p* =*0.023, Stroop: U* = *14.5, p* = *0.008, Digit Span: U* = *11, p* = *0.004*); with the exception of the CVLT, which was tested at baseline only due to practise effects.

### Ocular Motor Assessments

#### Prosaccades

There were no significant differences in latency between IIH and control groups for either the block PS task, or PS trials on the more cognitively complex AS-PS task, at any time-point (baseline, +3 months, +6 months). In addition, there was no significant group difference in error for PS trials in the AS-PS task, at any time-point.

#### Antisaccades

There was no significant difference in latency between IIH and control groups for the block AS task at baseline. In contrast, AS latencies were significantly prolonged for IIH patients on the more cognitively complex AS-PS task, compared to controls (*U* = *58, p* = *0.034*). In addition, IIH patients generated significantly more AS errors than controls on both the block (*U* = *7.5, p*< *0.001*) and AS-PS tasks (*U* = *44, p* = *0.007*).

At 3-months, AS latencies on the AS-PS task for IIH patients had reduced to within the normal range, with no statistically significant group difference (*U* = *37, p* = *0.056*). While AS error rates remained significantly higher for IIH patients at 3-months on the block AS task (*U* = *31.5, p* = *0.032)*, at 6 months and with only 5 patients available for testing, values had reduced to within the normal range (*U* = *22, p* = *0.583*).

### Effect of Clinical Variables on Cognitive Performance

Multivariate analyses demonstrated that IQ, headache, depression, anxiety, weight, and waist circumference did not predict any significant ocular motor or neuropsychology result at baseline. However, increased RNFL thickness predicted increased baseline AS error rate, both in the block [*F*_(3, 19)_ =*3.22, B* = *0.30, p* = *0.022*] and AS-PS task [*F*_(3, 20)_=*2.65, B* = *0.363, p* = *0.013*]. At 3-months, RNFL elevation did not clearly predict block AS error [*F*_(5, 12)_=*1.763, B* = *0.553, p* = *0.056*], although continued to predict AS-PS antisaccade error [*F*_(5, 13)_ = *3.186, B* = *0.589, p* = *0.008*].

Patient numbers were insufficient for multivariate analyses of 6-month data, or multivariate analyses of medication effect. When comparing means, interleaved anti-saccade task latencies appeared more prolonged in patients taking topiramate compared to those on acetazolamide (*404 vs. 326 ms, U* = *10, p* = *0.027*), however medications did not appear to impact performance on any other cognitive measure. In particular, the effects of topiramate on cognition may be more evident in larger cohorts, since there is a known association between topiramate and cognitive impairment (27).

## Discussion

We reveal a range of cognitive impairments in IIH patients both on conventional neuropsychological and novel ocular motor tests of cognition. At baseline, as well as 3 and 6 months, IIH patients performed more poorly than controls on the Stroop test, indicating poorer inhibition of cognitive interference (21), on the SDMT, indicating reduced cognitive processing speed (20), and on the Digit Span and CVLT, indicating reduced working memory (22, 23).

At baseline, IIH patients found it more difficult to inhibit a saccade toward a suddenly appearing stimulus (AS error), irrespective of task difficulty. However, eye movements made directly toward a visual stimulus (PS) were comparable to controls for both tasks, at all time-points. For the more cognitively complex interleaved saccade task, AS latencies were significantly prolonged for IIH patients, suggesting reduced cognitive processing speed (8). At 3 months, IIH patients exhibited partial improvement for AS error and normal anti-saccade latencies. In five patients followed for 6-months, all ocular motor results were similar to controls, although these results must be interpreted with caution given the significant cohort attrition over time.

Although IIH patients reported high rates of headache, anxiety and depression, none of these factors predicted performance on any neuropsychological or ocular motor measure, at any time-point. Notably, rates of headache and mood disturbances remained similar over time. However, increased AS error rates were associated with changes in structures involved in afferent visual transduction (i.e., increased RNFL thickness). Increased RNFL thickness was not associated with performance on any other cognitive measure. Further, RNFL thickness reduced significantly over time, in-line with observed improvements in ocular motor results. On the contrary, poorer performance on neuropsychological assessments for IIH patients persisted at 6 months, suggesting that cognitive functions less reliant on visual processing are independent of the clinical features of the disorder. It is plausible that subclinical visual processing changes may not impact conventional tests of cognition or perceived visual functioning, but may be revealed using more direct testing of the visual processing system using ocular motor tasks.

With the exception of a single case report (28), all prior studies have reported cognitive impairments in IIH (2, 29–33). Yri et al. in a study of 31 IIH patients, described decreased processing speed and reaction times as well as cognitive flexibility deficits (2). When considered in combination with our findings of prominent inhibitory control deficits and impaired cognitive flexibility, it is conceivable that impaired frontostriatal function may underlie cognitive impairments in IIH (34).

Frontostriatal circuits support cognitive functions and neuroanatomically encompass the frontal cortex, thalamus and basal ganglia (35). Three of five major frontostriatal circuits are thought to mediate non-motor, cognitively driven behaviours, namely the dorsolateral prefrontal, medial orbitofrontal and lateral orbitofrontal circuits (36). The dorsolateral pre-frontal cortex is primarily responsible for executive functioning, which is comprised of cognitive domains impaired in our IIH cohort, such as inhibitory control, processing speed and working memory (37). Although IIH pathophysiology is not fully understood, there is a complex yet well-established relationship between IIH and obesity, with the pathology of obesity postulated to affect striatal networks (38).

Weight gain and obesity increase the risk of IIH, while modest amounts of weight loss can lead to disease resolution (39). In our cohort, weight and waist circumference did not predict cognitive performance. Similarly, Yri et al. found that IIH patient body mass index (BMI) did not predict cognitive performance (2) suggesting that cognition appears to be influenced by the presence of, rather than the magnitude of weight excess in IIH. However, obesity by itself, or when present in other neurological diseases, has been associated with a number of adverse cognitive outcomes (40, 41) and larger cohorts may be needed to resolve this association. Functional Magnetic Resonance Imaging (fMRI) suggests that obesity and binge-eating disorder is associated with decreased striatal responses to food, yet heightened frontostriatal responses to food cues (42). This supports the hypothesis that frontostriatal changes in obesity may facilitate decreased inhibitory control (43).

A further potential pathophysiological factor in obesity and IIH-related cognitive changes is the metabolically activity of adipose tissue, that can produce a range of adipokines and inflammatory cytokines (44). Such an inflammatory milieu may contribute to cognitive impairment by interfering with neuronal network function. This has been demonstrated in other conditions such as multiple sclerosis, where obesity independently contributes to cognitive dysfunction as assessed by clinical testing, biomarkers, and MRI (45). Obesity-related systemic inflammation as well as increased mechanical strain on frontostriatal networks from raised intracranial pressure may therefore both contribute to cognitive dysfunction in IIH (46). Since IIH patients exhibited no PS deficits, basic visual processing appears intact. Visual processing changes must therefore relate to the interaction of signals from the afferent visual pathway and widely distributed cognitive networks that are utilised in generating an AS (47). This is clinically relevant, since high rates of AS errors are associated with decreased concentration and high distractibility (48).

Surprisingly, we found no correlation between any ocular motor measure and neuropsychology test results in our study. This was especially surprising for tests of inhibitory control like the AS task and Stroop test. However, the Stroop test and AS task assess inhibitory control differently. Unlike the AS task, there are no absolute penalties for errors in the Stroop test (21), which records errors as a correct/incorrect binary variable. The Stroop test and AS task are also timed differently; the Stroop test is concluded when a time limit is reached, while the AS task measures the time taken to complete a task. While we might expect a relationship between saccade latency and deficits revealed by neuropsychological testing, as has been reported previously (49), this is conceivably confounded by high error rates due to impaired inhibitory control, excluding a large number of trials from latency analyses for our IIH cohort.

Our study was limited by a relatively homogenous group of IIH patients, with mild to moderate clinical characteristics. Longitudinal testing was impacted by Covid19 restrictions, and a larger, more heterogenous cohort of IIH patients would help to confirm trends and clarify clinical associations identified in our study. Ideally controls would be weight matched to the IIH group in addition to being age and sex matched, since cognitive impairments may be associated with obesity (43). Further, ocular motor tasks less reliant on inhibitory control would be useful in future IIH research, identifying impairments obscured by the high AS errors in our study.

While our results were largely consistent with previous studies in IIH patients, we also revealed a unique subclinical cognitive profile in IIH, that elucidates the difficulties some IIH patients have with maintaining employment and engaging in lifestyle alterations (2, 39). Although it is increasingly acknowledged that cognitive impairments are likely in IIH, cognitive screening is absent from management guidelines (50). The Mini Mental State Examination (MMSE) has been proposed as a screening test (32), however it lacks sensitivity (24). The SDMT is quick, simple to administer and easy to score (20), and could be considered as an alternative screening test for IIH-specific cognitive changes, such as reduced information processing speed (2).

Weight, waist circumference, anxiety, depression, and headache do not appear to underlie cognitive impairments in mild to moderate IIH but need to be studied in larger cohorts. Here, RNFL elevation was associated with ocular motor deficits, that might represent subclinical change in visual processing and cognition and future inclusion of more severely affected IIH patients would be of interest. Exploration of frontostriatal pathways, given impairments of inhibitory control, processing speed, and working memory, may provide insights into IIH pathophysiology. Our work adds to the importance of the inclusion of cognitive screening in IIH management to enable targeted neurorehabilitation and employment support, leading to improved patient care.

## Statistical Analysis

Statistical analysis was performed by WW and MC.

## Data Availability Statement

The raw data supporting the conclusions of this article will be made available by the authors, without undue reservation.

## Ethics Statement

The studies involving human participants were reviewed and approved by Alfred Health Human Research and Ethics Committee. The patients/participants provided their written informed consent to participate in this study.

## Disclosure

NS served on advisory boards for Merck and Roche, and has received travel support from Biogen.

AV served on advisory boards and receives unrestricted research grants from Novartis, Biogen, Merck and Roche. She has received speaker's honoraria and travel support from Novartis, Roche, and Merck. She receives grant support from the National Health and Medical Research Council of Australia and MS Research Australia.

OW receives discretionary research funding from Merck, and is an Associate Editor for Frontiers in Neuro-Ophthalmology.

JF receives funding for other research from Biogen and Genzyme.

## Author Contributions

WW: recruitment, data acquisition, data interpretation, drafting, and revision of manuscript. MC: data acquisition, data interpretation, study supervision, and revision of the manuscript. NS: recruitment and revision of the manuscript. AV, OW, and JF: design and conceptualisation of the study, study supervision, and revision of the manuscript. All authors contributed to the article and approved the submitted version.

## Conflict of Interest

The authors declare that the research was conducted in the absence of any commercial or financial relationships that could be construed as a potential conflict of interest.

## Publisher's Note

All claims expressed in this article are solely those of the authors and do not necessarily represent those of their affiliated organizations, or those of the publisher, the editors and the reviewers. Any product that may be evaluated in this article, or claim that may be made by its manufacturer, is not guaranteed or endorsed by the publisher.
